# Decreased frequency of Th22 cells and IL-22 cytokine in kidney transplant patients with active cytomegalovirus infection

**DOI:** 10.1186/s12865-023-00555-2

**Published:** 2023-07-04

**Authors:** Yashgin Hassanzadeh, Ramin Yaghobi, Parviz Pakzad, Bita Geramizadeh

**Affiliations:** 1 Department of Microbiology, North Tehran Branch, Islamic Azad University, Tehran, Iran; 2 Shiraz Transplant Research Center, Shiraz University of Medical Sciences, Shiraz, Iran

**Keywords:** CD4^+^ T cells, Human cytomegalovirus, IL-22, Kidney transplantation, Th22

## Abstract

**Background:**

The immunity of CD4^+^ T cell subsets against human cytomegalovirus (HCMV) is considerable due to their essential role in controlling the infection in transplant individuals. Previously explained CD4^+^ subsets such as T helper (Th) 1 have been proven to have a protective role against HCMV infection, while the role of the recently identified Th22 subset has not been described yet. Here, the frequency changes of Th22 cells and the IL-22 cytokine production were investigated in kidney transplant recipients with and without HCMV infection.

**Methods:**

Twenty kidney transplant patients and ten healthy controls were enrolled in this study. Patients were categorized into HCMV + and HCMV- groups based on the HCMV DNA real-time PCR results. After isolating CD4^+^ T cells from PBMCs, the phenotype (CCR6^+^CCR4^+^CCR10^+^) and cytokine profile (IFN-γ^−^IL-17^−^IL-22^+^) of Th22 cells were analyzed by flow cytometry. The gene expression of Aryl Hydrocarbon Receptor (AHR) transcription factor was analyzed by real-time PCR.

**Results:**

The phenotype frequency of these cells was lower in recipients with infection than in those without infection and healthy controls (1.88 ± 0.51 vs. 4.31 ± 1.05; *P* = 0.03 and 4.22 ± 0.72; *P* = 0.01, respectively). A lower Th22 cytokine profile was observed in patients with infection than in the two other groups (0.18 ± 0.03 vs. 0.20 ± 0.03; *P* = 0.96 and 0.33 ± 0.05; *P* = 0.04, respectively). AHR expression was also lower in patients with active infection.

**Conclusions:**

Overall, this study for the first time suggests that the reduced levels of Th22 subset and IL-22 cytokine in patients with active HCMV infection might indicate the protective role of these cells against HCMV.

## Background

Human cytomegalovirus (HCMV) is the most opportunistic recurrent virus in kidney transplantation, causing viremia, disease, or even organ rejection [1–4]. As central orchestrators of the immune system, CD4^+^ T cells have an important role in HCMV infection [2]. The frequency of CD4^+^ T cells specific for HCMV is inversely correlated with the virus proliferation and DNA load in kidney transplant recipients [5]. Also, CD4^+^ T cells’ ongoing responses against HCMV are associated with the control and clearance of infection. Thus, understanding their function mechanism is essential to designing new therapies [6].

The CD4^+^ T cells are heterogenous and exert their direct antiviral function (e.g., cytolytic activity) along with their traditional helper function (e.g., enhancement of CD8^+^ T cells and B cells antibody production) via their various subsets [7, 8]. When these cells encounter a pathogen, they differentiate into functionally distinct subsets, including T helper (Th) 1, Th2, Th17, Th9, Th22, Tfh, and Treg cells determined by their master transcription factor and signature cytokines [2, 6, 7, 9–11].

The protective role of Th1 cells against HCMV has been shown, and their frequency is associated with infection occurrence. Th1 cells producing TNF-α, IFN-γ, and IL-2 were reduced during HCMV active infection in Solid Organ Transplant (SOT) recipients, e.g., kidney and liver [12–15]. In contrast, evidence shows that Th2 cells producing IL-4 may exacerbate infection by reducing Th1 immunity or transmitting HCMV to monocytes [9, 13, 14, 16, 17]. The Tfh cells producing IL-21 are related to HCMV control in SOT patients via increasing the neutralizing and IgG antibodies. An increased level of these cells is associated with HCMV clearance [18–20]. Moreover, the increased frequency of Treg cells producing TGF-β and IL-10 is associated with the progressive proliferation of the virus. Their suppressive cytokines can reduce effector CD4^+^ and CD8^+^ T cells [21–24]. However, the role of recently identified CD4^+^ T cell subsets, i.e., Th22 and Th9, against the HCMV infection, has not been evaluated yet.

Knowledge about the role of Th22 cells in viral infection is little, and to the best of our knowledge, no studies have been conducted on HCMV infection. Th22 cells produce IL-22 as a signature cytokine and express Aryl Hydrocarbon Receptor (AHR) as a lineage-specific transcription factor. Depending on the inherent infection factors, they have divergent protective and pathologic roles in viral infections [11, 25, 26]. TNF-α and IL-6 cytokines induce the naive CD4^+^ cells’ differentiation to Th22 with the phenotype of C-C Chemokine Receptor (CCR) 4^+^ CCR6^+^ CCR10^+^ [27, 28].

IL-22 interaction with its receptor IL-22R subsequently activates of JAK1 and Tyk2 and phosphorylation of STAT1, STAT3, and STAT5 to exert antiviral and tissue protective function [26, 29, 30]. For example, in HIV infection, it is observed that the increased amounts of Th22 cells are associated with resistance to infection and IL-22 has a protective role in mucosal sites [26, 31]. Contrary to the previous case, secreted IL-22 in West Nile virus infection mediates viral dissemination by recruiting neutrophils in infection sites [25]. A dual opposite function has also been reported for IL-22 in defense against HBV [32].

Therefore, the present study aimed to evaluate the presence and frequency of Th22 cells and their signature cytokine, IL-22, as a first step to shedding light on their functional nature in kidney transplant patients with and without HCMV infection. Besides, the frequency of Th17 cells and IL-17 cytokine were reported. We showed that Th22 cells and IL-22 decreased in recipients with active infection, parallel to a decrease in Th17 cells.

## Results

### Patient characteristic

A total of 30 individuals (19 males and 11 females) consisting of 10 healthy controls and 20 kidney transplant patients were enrolled in this study. The healthy HCMV seropositive (IgG 6.25 ± 0.84 IU/ml; range, 0.86–8.40) control group consisted of five (50%) males and five (50%) females with a mean age of 38.70 ± 3.00, ranging from 30 to 52 years old. Of the 20 patients, 10 were classified as HCMV+, consisting of eight (80%) males and two (20%) females (age range, 32–69 years old) with a viral load from 1 × 10^4^ to 4 × 10^6^ copies/ml. Another 10 patients were classified as HCMV-, consisting of six (60%) males and four (40%) females (age range, 23–66 years old). All the enrolled individuals were positive for HCMV IgG. Fifteen patients who experienced rejection received intravenous methylprednisolone (500 mg) for three days. Out of these fifteen patients, ten patients received intravenous antithymocyte globulin (ATG) (Thymoglobulin) 75 mg daily for 3 to 4 days, and two patients received 20 mg basiliximab daily for four days plus ATG. Patients had ABO-compatible transplantation. Detailed information on kidney transplant patients is outlined in Table 1.

**Table 1:** Demographic and clinical characteristics of kidney transplant patients

Variable	SOT patients (total)	HCMV-^**a**^ (n^**c**^=10)	HCMV+^**b**^ (n = 10)	*P* value^*^
Age (Year)^**i**^	45.75 ± 3.58	38.10 ± 4.80	53.40 ± 4.27	**0.02**
Sex ^**j**^				0.062
Male female	14 (70%) 6 (30%)	6 (60%) 4 (40%)	8 (80%) 2 (20%)	
HCMV IgG (IU/ml)^**i**^	8.08 ± 0.14	7.91 ± 0.26	8.25 ± 0.06	1.00
Living donor Tx ^**d j**^ Related ^**j**^ Unrelated ^**j**^	5 (25%) 2 (10%) 3 (15%)	3 (30%) 1 (10%) 2 (20%)	2 (20%) 1 (10%) 1(10%)	
Dialysis prior to Tx ^**j**^	12 (60%)	5 (50%)	7 (70%)	
Types of dialysis ^**j**^				
HD^**e**^ PD^**f**^	11 (55%) 1 (5%)	4 (40%) 1 (10%)	7 (70%) 0	
Time on dialysis (Months)^**i**^	20.18 ± 3.72	15.20 ± 6.61	24.33 ± 3.76	0.23
Rejection ^**j**^ Acute rejection Chronic rejection	15 (75%) 9 (45%) 6 (30%)	8 (80%) 5 (50%) 3 (30%)	7 (70%) 4 (40%) 3 (30%)	
Induction therapy ^**j**^ ATG ^**g j**^ (1.5 mg/kg) ATG + Basiliximab ^**j**^ (20 mg)	12 (60%) 10 (50%) 2 (10%)	6 (60%) 6 (60%) 0	6 (60%) 4 (40%) 2 (20%)	
ESRD^**h**^ etiology ^**j**^				
Hypertension Diabetes mellitus Glomerulonephritis	16 (80%) 2 (10%) 1 (5%)	9 (90%) 0 1 (10%)	7 (70%) 2 (20%) 0	
Clinical parameters^**i**^				
BUN (mg/dL) Cr (mg/dL) Na (mmol/L) K (mmol/L) Ca (mg/dL) P (mg/dL) Mg (mmol/L) Uric A. (mg/dL) FBS (mg/dL) WBC (×10^3^/mm^3^) Hb (g/dL) HCT (%) Plt (×10^9^/L)	37.75 ± 3.40 3.00 ± 0.40 137 ± 1.07 4.19 ± 0.16 8.16 ± 0.13 4.04 ± 0.14 1.81 ± 0.06 6.58 ± 0.33 100.10 ± 6.97 8.12 ± 0.53 9.89 ± 0.46 30.77 ± 1.39 195.20 ± 25.25	32.50 ± 4.50 2.10 ± 0.32 139.30 ± 1.30 4.37 ± 0.24 8.11 ± 0.15 3.89 ± 0.15 1.87 ± 0.08 6.30 ± 0.53 87 ± 5.41 9.10 ± 0.60 9.59 ± 0.49 30.01 ± 1.62 236.50 ± 41.60	43.00 ± 4.73 3.90 ± 0.64 136.20 ± 1.62 4.01 ± 0.23 8.22 ± 0.21 4.20 ± 0.23 1.76 ± 0.09 6.86 ± 0.40 113.20 ± 11.75 7.15 ± 0.80 10.20 ± 0.79 31.53 ± 2.33 153.90 ± 24.12	0.08 **0.03** 0.29 0.51 0.66 0.24 0.26 0.44 **0.04** 0.09 0.75 0.80 0.31

^**a**^HCMV-: patients without HCMV infection; ^**b**^HCMV+: patients with active HCMV infection; ^**c**^n: number; ^**d**^Tx: transplantation; ^**e**^HD: hemodialysis; ^**f**^PD: peritoneal dialysis; ^**g**^ATG: Anti-thymocyte globulin; ^**h**^ESRD: end stage renal disease; ^**i**^: Results expressed as mean ± SEM; ^**j**^: Results expressed as number (% of patients in the corresponding group); ^*^P ≤ 0.05.

### Th22 phenotypic profiling

**Fig. 1:**
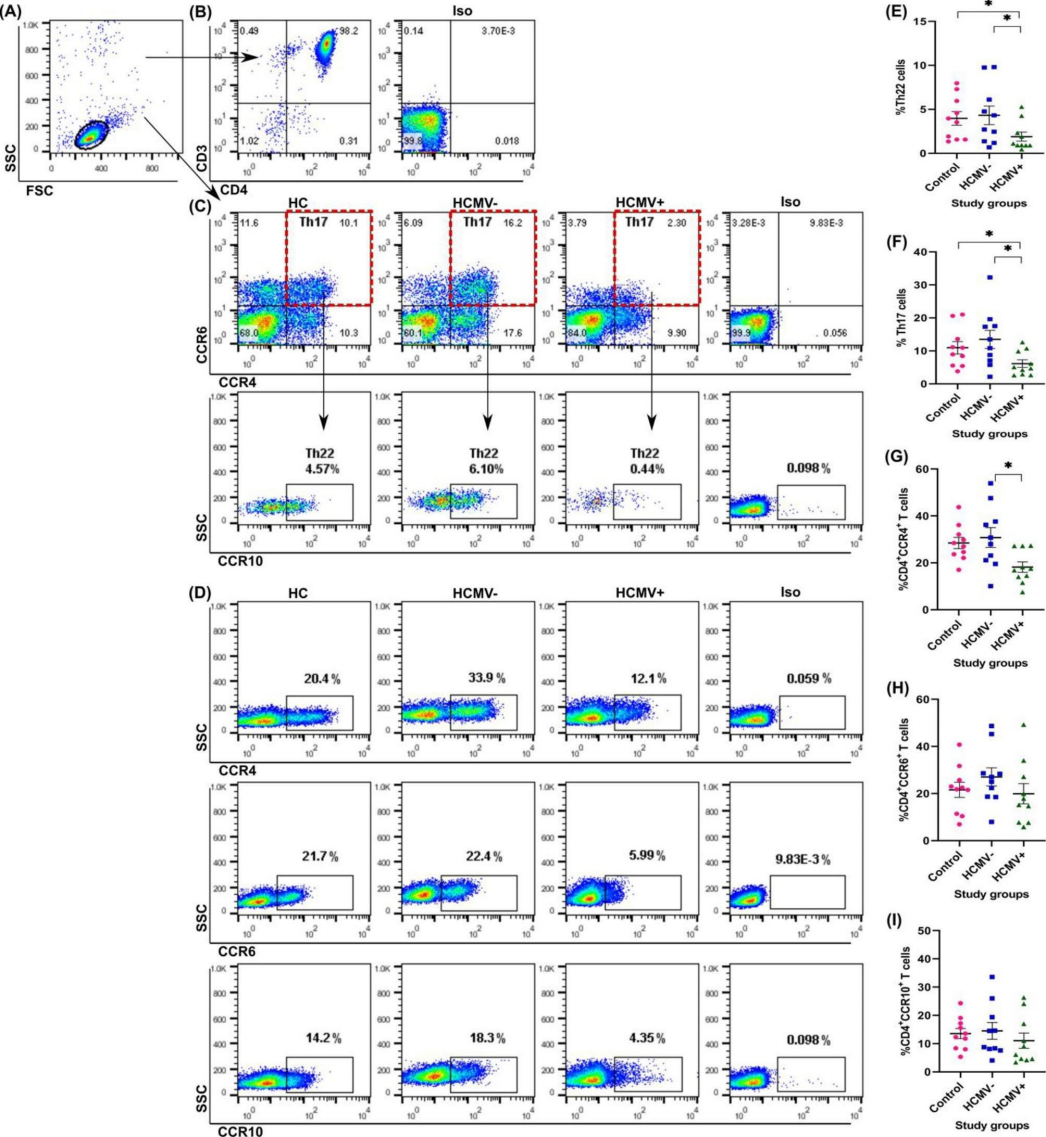
The phenotype frequency of Th22 cells in study groups. **A**. The isolated CD4^+^ T cells against the side and forward scatter to gate on the lymphocyte population. **B**. The purity analysis of isolated CD4^+^ T cells and gating strategy by flow cytometry. The frequency of ≥ 95% double positive CD3^+^CD4^+^ T cells was considered a pure population. **C**. Gating strategy to measure the phenotype frequency of Th22 cells (CCR6^+^CCR4^+^CCR10^+^) and Th17 cells (CCR6^+^CCR4^+^). First, the CCR6^+^CCR4^+^ double positive cells (red dashes) were gated for each study group, and then the CCR10^+^ population was measured against side scatter. Red dashes determine the population of Th17 cells (CCR6^+^CCR4^+^). Isotype controls were used to set gates. **D**. The frequency of each surface chemokine receptor was evaluated against the side scatter in three study groups. Isotype controls were used to set gates. **E**. The statistical comparison of Th22 cells phenotype frequencies (CCR6^+^CCR4^+^CCR10^+^) between study groups. **F**. The statistical comparison of Th17 cell phenotype frequencies (CCR6^+^CCR4^+^) between study groups. **G, H, I**. The statistical comparison of each surface chemokine receptor frequency in different study groups. SSC, side scatter; FSC, forward scatter; Iso, isotype control; HC, healthy control; HCMV-, kidney transplant patients without active infection; HCMV+, kidney transplant patients with active infection; *, statistically significant at *P* ≤ 0.05

The gating strategy for evaluating the Th22 phenotype is shown in Fig. 1. After gating on the isolated CD4^+^ T cells, their purity was analyzed by measuring double positive CD3^+^CD4^+^ T cells. The Th22 phenotype (CCR6^+^CCR4^+^CCR10^+^) was analyzed first by gating on double positive CCR6^+^CCR4^+^ T cells population and then on the CCR10^+^ T cells population. The data showed that the Th22 phenotype was significantly lower in HCMV + patients than in HCMV- patients and healthy individuals (1.88 ± 0.51 vs. 4.31 ± 1.05; *P* = 0.03 and 4.22 ± 0.72; *P* = 0.01, respectively). However, the frequency of Th22 in HCMV- patients was similar to healthy individuals (4.31 ± 1.05 vs. 4.22 ± 0.72; *P* = 0.81). Similarly, the mean frequency of Th17 phenotype (CCR6^+^CCR4^+^) was significantly lower in HCMV + patients than in negative ones and healthy controls (6.42 ± 1.13 vs. 13.47 ± 2.75; *P* = 0.02 and 11.50 ± 1.79; *P* = 0.03, respectively). The Th17 frequency was higher in HCMV- patients than in control individuals but was not statistically significant (13.47 ± 2.75 vs. 11.50 ± 1.79; *P* = 0.93). Regarding each of the CCR4, CCR6, and CCR10 chemokine receptors, after analyzing them against side scatter, their frequencies showed lower amounts in HCMV + patients than in HCMV- and control groups. The distribution of CD4^+^CCR6^+^ and CD4^+^CCR10^+^ T cells showed no statistical difference between the study groups. However, CD4^+^CCR4^+^ T cells showed significantly lower frequency in HCMV + patients than in HCMV- ones (18.99 ± 2.07 vs. 30.78 ± 4.22; *P* = 0.02). It is while there was no difference between the HCMV- and healthy control groups regarding CD4^+^CCR4^+^ T cells (30.78 ± 4.22 vs. 28.32 ± 2.39; *P* = 1.00). We then evaluated the frequency phenotype changes of Th22 and Th17 cells between patients who received induction therapy and those who did not. There were no differences between these subgroups in both HCMV + and HCMV- groups.

### Th22 cytokine profiling

**Fig. 2:**
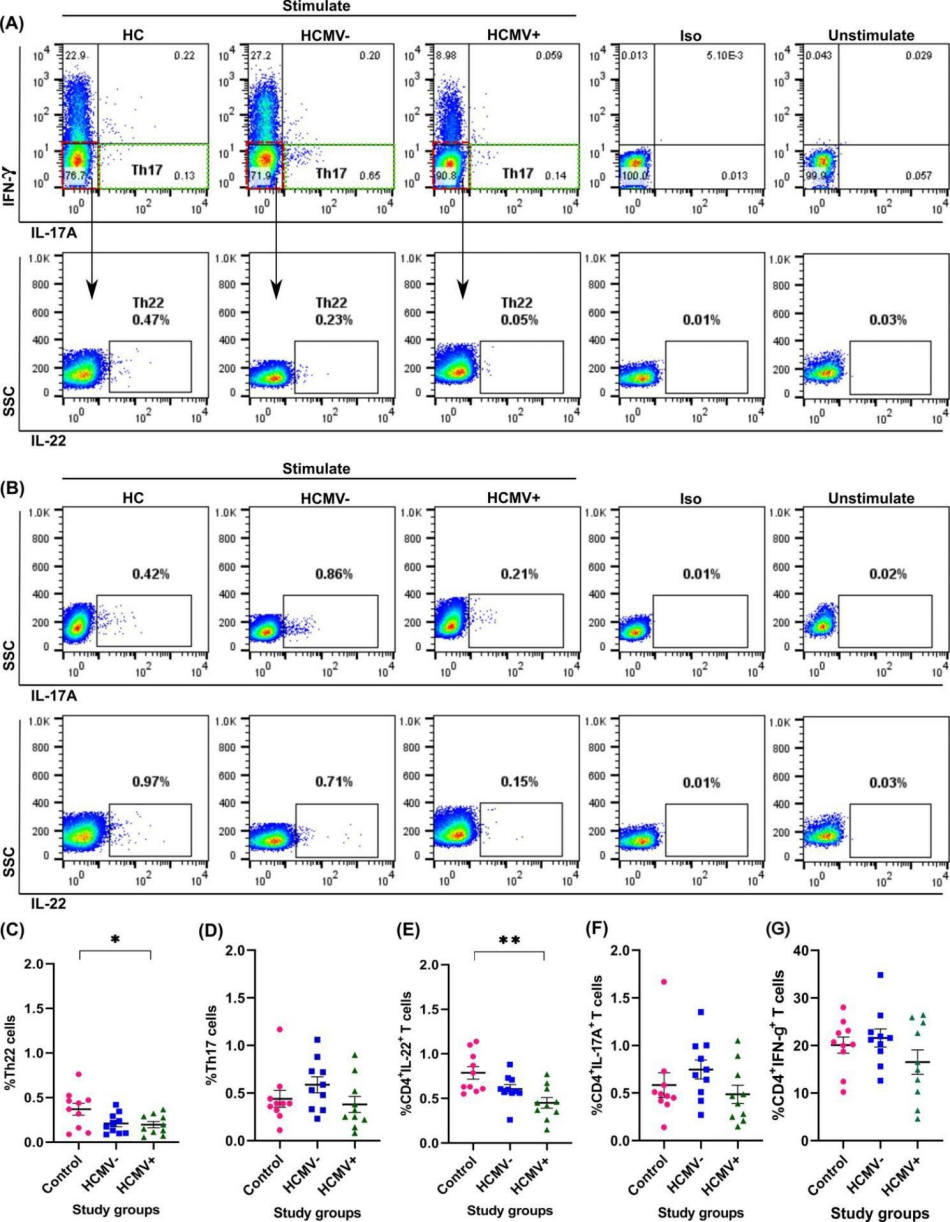
The frequency of Th22 cytokine profile in study groups. **A**. gating strategy to measure the frequency of Th22 cells (IFN-γ-IL-17 A-IL-22+) and Th17 cells (IFN-γ-IL-17 A+). The green dashes show the frequency of Th17 cells. The IFN-γ-IL-17- double negative cells (red dashes) were gated; then IL-22 + cells were measured. **B**. The frequency of IL-22 and IL-17 A cytokines in three study groups. **C**. Comparing the percentage of Th22 cytokine profile (IFN-γ-IL-17 A-IL-22+) in the study groups. **D**. Comparing the percentage of Th17 cytokine profile (IFN-γ-IL-17 A+) in the study groups. **E, F, G**. Comparing the percentage of IL-22, IL-17 A, and IFN-γ cytokines in the study groups. SSC, side scatter; FSC, forward scatter; Iso, isotype control; HC, healthy control; HCMV-, kidney transplant patients without active infection; HCMV+, kidney transplant patients with active infection; *, statistically significant at *P* ≤ 0.05; **, statistically significant at *P* ≤ 0.01

Intracellular staining of IFN-γ, IL-17, and IL-22 was performed on the isolated CD4^+^ T cells to determine the Th22 frequency in the study groups (Fig. 2). The Th22 cells (IFN-γ^−^IL-17^−^IL-22^+^) were lower in HCMV + than in HCMV- patients and significantly lower in healthy individuals (0.18 ± 0.03 vs. 0.20 ± 0.03; *P* = 0.96 and 0.33 ± 0.05; *P* = 0.04, respectively). These cells were also lower in HCMV- patients than in the healthy control group (0.20 ± 0.03 vs. 0.33 ± 0.05; *P* = 0.08), but not statistically significant. The Th17 frequency was lower in the HCMV + group than in the negative and healthy groups (0.39 ± 0.08 vs. 0.59 ± 0.08; *P* = 0.23 and 0.45 ± 0.08; *P* = 0.88, respectively). The cytokine profile of these cells was slightly higher in HCMV- patients than in the healthy control group (0.59 ± 0.08 vs. 0.45 ± 0.08; *P* = 0.46) but not statistically significant. The frequency of IL-22 cytokine was lower in HCMV + patients than in HCMV- patients and significantly lower compared to healthy control group (0.41 ± 0.04 vs. 0.58 ± 0.04; *P* = 0.07 and 0.75 ± 0.06; *P* = 0.001, respectively). IL-17 cytokine was lower in the HCMV + group than in HCMV- and healthy ones (0.48 ± 0.09 vs. 0.75 ± 0.10; *P* = 0.21 and 0.60 ± 0.12; *P* = 0.74, respectively). Moreover, the frequency of IFN-γ cytokine was lower in HCMV + individuals than in HCMV- and healthy ones (17.08 ± 2.58 vs. 21.58 ± 1.89; *P* = 0.26 and 20.91 ± 1.29; *P* = 0.38, respectively) although the differences were not significant. The cytokine profile comparison of Th22 and Th17 cells between patients who did not receive induction therapy and those who did showed no differences in both HCMV + and HCMV- groups.

### Correlation between Th22 and Th17 cells in HCMV + and HCMV- patients

**Fig. 3:**
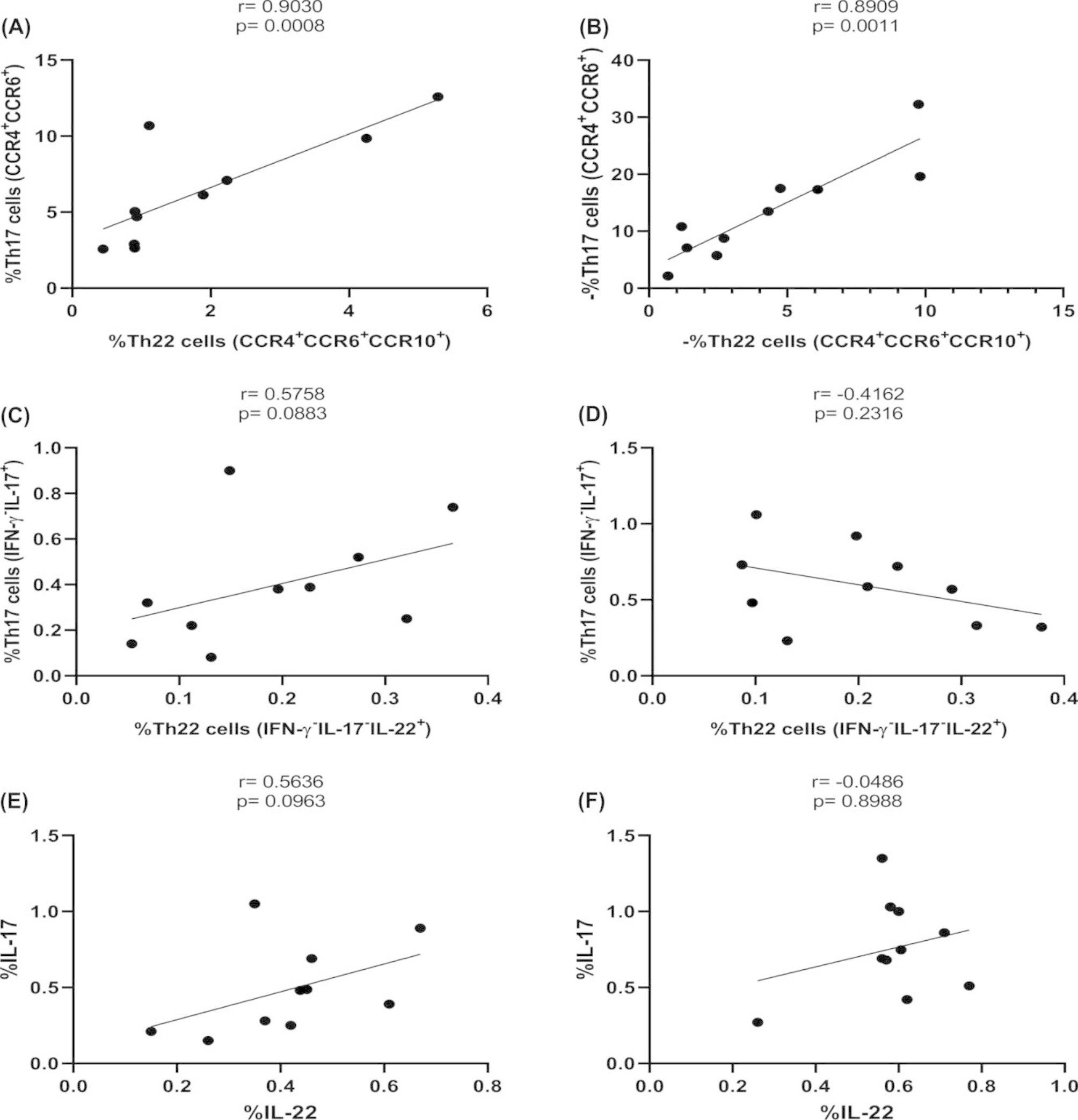
The correlation analysis between the frequency of Th22 and Th17 cells and their canonical cytokines. **A**. Correlation between Th22 and Th17 phenotypes in HCMV + patients. **B**. Correlation between Th22 and Th17 phenotypes in HCMV- patients. **C**. Correlation between Th22 and Th17 cytokine profiles in HCMV + patients. **D**. Correlation between Th22 and Th17 cytokine profiles in HCMV- patients. **E**. Correlation between IL-22 and IL-17 A cytokines in HCMV + patients. **F**. Correlation between IL-22 and IL-17 A cytokines in HCMV- patients

The Spearman analysis demonstrated that the frequency of the Th22 phenotype was positively correlated with the Th17 phenotype in HCMV + and HCMV- patients (r = 0.903, *P* = 0.0008 and r = 0.890, *P* = 0.001, respectively). However, their frequency obtained by measuring intracellular cytokines was not related to the frequency of Th17 in both groups (r = 0.575, *P* = 0.088 and r= -0.416, *P* = 0.2316, respectively) (Fig. 3). The IL-22 cytokine also was not correlated with IL-17 cytokine in either group (r = 0.563, *P* = 0.096 and r= -0.048, *P* = 0.898, respectively).

### Determination of the cut-off value of Th22 and Th17 phenotypes

**Fig. 4:**
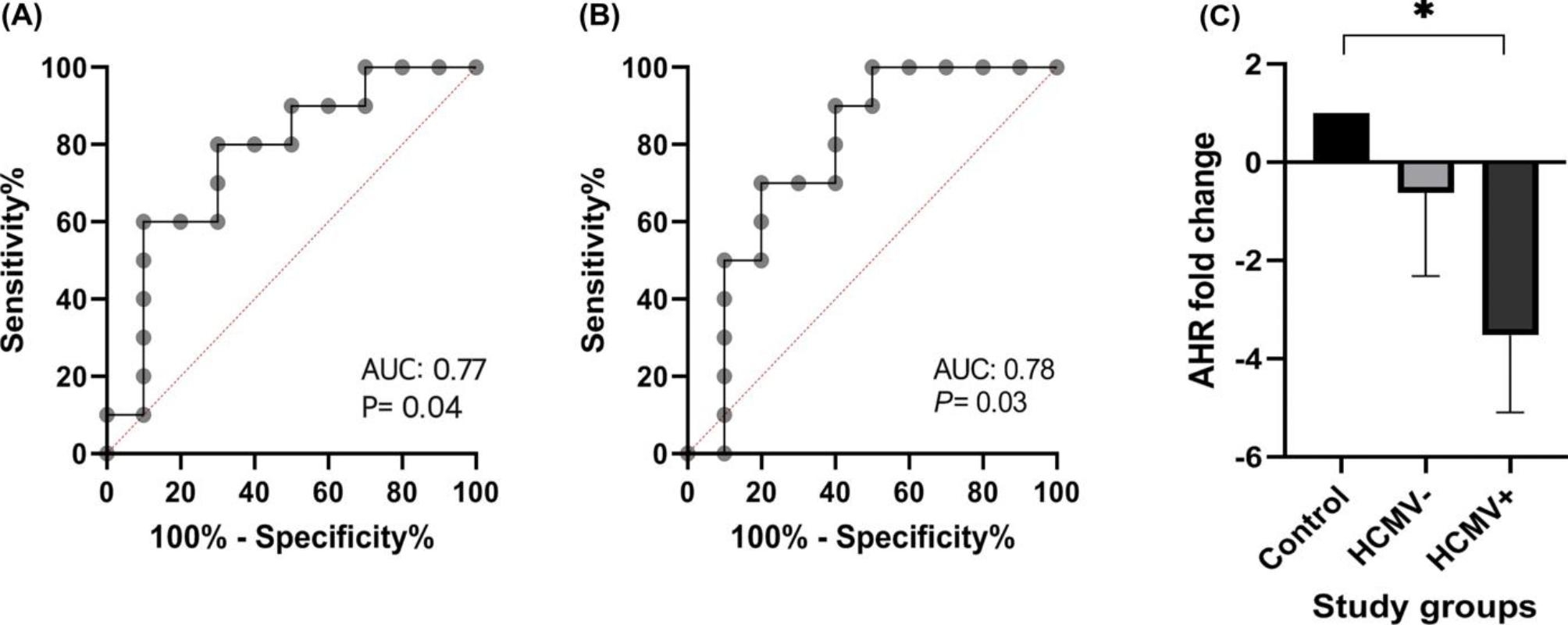
ROC curve analysis of Th22 and Th17 phenotype frequency and the expression of AHR transcription factor. The sensitivity and specificity of different cut points were analyzed to generate the ROC curves. The outcome of interest for both Th22 and Th17 is the prediction of HCMV infection. **A**. ROC curve of the phenotype frequency of Th22 cells. **B**. ROC curve of the phenotype frequency of Th17 cells. **C**. Statistical analysis of the expression of AHR transcription factors in the study groups

Regarding the HCMV infection in transplant patients with decreased levels of Th22 and Th17 phenotypes, the ROC curve analysis showed that the frequency of Th22 phenotype in blood had an 80% sensitivity and 70% specificity at a cut-off value of ≤ 2.34% (AUC = 0.77, 95% CI = 0.55–0.98, *P* = 0.04) to predict infection (Fig. 4). The frequency of Th17 phenotype ≤ 7.08% could also predict HCMV infection with 70% sensitivity and 80% specificity (AUC = 0.78, 95% CI = 0.56–0.99, *P* = 0.034).

### AHR expression

The gene expression of AHR was significantly lower in HCMV + patients than in healthy controls (-3.52 ± 1.56 vs. 1.00 ± 0.00; *P* = 0.04) and lower than HCMV- patients (-3.52 ± 1.56 vs. -0.62 ± 1.69; *P* = 0.43). Moreover, AHR expression was lower in HCMV- patients than in the healthy control group (-0.62 ± 1.69 vs. 1.00 ± 0.00; *P* = 0.61). However, the difference was not significant.

## Discussion

Th22 cells and their cytokine, IL-22, have been shown to protect against various bacterial and viral infections. However, there was no data about these cells’ role in HCMV infection. Thus, we have studied the frequency and functionality of the Th22 subset by staining a combination of chemokine receptors (CCR4^+^CCR6^+^CCR10^+^) and intracellular cytokines (IFN-γ^−^IL-17 A^−^IL-22^+^) and the expression of AHR transcription factor in kidney transplant patients with HCMV reactivation. In addition, the frequency of Th17 cells was studied.

Our results demonstrated that these cells were lower in HCMV + kidney transplant patients than in HCMV- patients and healthy controls. Lower frequencies of IL-22 and AHR expression were also observed. Similar results were observed for the frequency of Th17 cells and IL-17. The ROC curve analysis showed that the phenotype frequency of both Th22 and Th17 cells could be used to predict HCMV infection. The area under the curve was quite the same for both subsets. Our findings provide new data about the possible role of Th22 cells in the immune response against HCMV infection and open an area for further research. Simultaneous assessment of cell phenotype and functionality could accurately determine the status of Th22 in defeating the infection. These cells could be used to predict HCMV infection in kidney transplant patients, but further validation is needed.

We believe there is no study on the frequency of Th22 cells and IL-22 production in HCMV infection. Regarding other viral infections, studies have reported IL-22 production by T cells with an antiviral protective role. The increased levels of Th22 cells were observed in acute viral myocarditis induced by coxsackievirus B3 (CVB3) with a protective role [28]. Repeatedly HIV-1 exposed but uninfected individuals have been shown to overproduce IL-22 cytokine by CD4^+^ T cells with a possible protective role through acute phase proteins such as serum amyloid A [31, 33]. Indeed, increased systemic levels of IL-22 were related to low HIV replication in vitro [33]. Page et al. reported Th22 cell reduction in untreated HIV-1 infected patients [34]. In our study, we observed the reduced frequency of Th22 in both phenotype and cytokine profiles in HCMV + patients, suggesting their possible antiviral role in HCMV- patients.

In a murine cytomegalovirus study, different immune cells, such as T and NK cells, produced IL-22 in response to the infection. The secreted IL-22 was determined to restrict MCMV function by recruiting neutrophils. IL-22 induced the production of CXCL1 chemokine to recruit neutrophils at the infection sites. These neutrophils show their anti-MCMV function through the TRIAL/TRAILR pathway [26]. In addition, the in vivo increased expression of IL-22 mRNA has been reported [30]. As a convenient model for HCMV in host immunity and pathogenesis, this study may bring the same antiviral properties in mind for HCMV. However, it has been revealed that HCMV facilitates its dissemination during acute infection through neutrophil recruitment by encoding the homolog of CXCL1 (UL146). Notably, in immunosuppressed individuals, neutropenia was proposed as a risk factor for herpesvirus infection [26]. Therefore, further investigation is needed.

The reduction of Th22 cells in this study could result from the progressive proliferation of HCMV in our patients, as the viral load for all of them was more than 1 × 10^4^ copies/ml. Thus, due to high viral load and possible increased immunopathology of immune cells, the immune modulatory cells such as Treg cells may disrupt immune responses by TGF-β and IL-10 production [21]. In MCMV infection, a high viral load was observed in mice lacking IL-22 [26].

TGF-β inhibits the differentiation of Th22 from naïve CD4^+^ T cells [20, 35]. Moreover, virus-encoded IL-10 homolog, capable of inducing host IL-10, synergizes with this process to make it worse and modulates the immune system [36, 37]. IL-10 maintains the differentiation of monocytes to macrophages, a proper place for the proliferation of the virus, and up-regulates HCMV immediate-early genes to attenuate antiviral responses [38, 39]. It has been proposed that the decreased ratio of Th22/Treg may contribute to immune deficiency in HIV-1 infection [31]. Therefore, it could be a possible reason for the Th22 and Th17 reduction in our study, which needs further investigation.

It may also bring to mind that the reduction in these cells in HCMV + patients could be due to immunosuppression therapy that transplanted patients received. It is evident that immunosuppression affects the frequency and composition of CD4^+^ T cell subsets, including a reduction in the IFN-γ producing Th1 and circulating follicular regulatory T cells (cTfr) [40, 41]. To the best of our knowledge, the effect of immunosuppressive drugs on Th22 cells and cytokine has not been studied well. It may be due to little knowledge about the involvement of these cells in organ rejection, as Claeys et al. and Liu et al. mentioned [42, 43]. The only difference between HCMV- patients and the control group in this study was the receipt of the immunosuppressive drugs by the patients. Our results showed no differences in the frequency phenotype of Th22 and Th17 cells between these two groups. However, both HCMV + and HCMV- kidney transplanted patients were under the same condition and received immunosuppressive drugs. So, reduced Th22 and Th17 phenotype frequency in the HCMV + group compared to the HCMV- one may bring the hypothesis that HCMV infection may impact on these cell populations.

The role of Th22 cells in viral infections depends on the context and is less well-defined [25, 26, 28, 33]. The factors encoded by a specific pathogen likely affect the outcome of Th22 responses [25]. Moreover, it has been reported that IL-22 can have paradoxical functions in a distinct viral infection. As demonstrated before, IL-22 can promote the chemokine-induced recruitment of proinflammatory Th17 cells in HBV infection, which result in inflammation and liver damage [33]. However, IL-22 alone can protect hepatocytes from apoptosis in viral hepatitis-induced liver damage [44].

IL-22 mainly functions by synergizing with other cytokines and chemokines to recruit immune cells [33]. For example, the signaling of IL-22 through interaction with IL-22R can modulate the production of cytokines such as IFN-γ, IL-17, TNF-α, IL-1β, and IL-6 [28, 44]. It is important to know which signaling pathways and cytokines are induced by Th22 cells and IL-22 to predict their exact role in a particular viral infection [33]. In our study, both IL-22 and IFN-γ were reduced in HCMV + patients. It may be related to the immunological status of these patients, indicating the defect of Th1 immunity, a crucial factor for resolving the infection.

Different studies have shown a positive correlation between the frequency of Th17 and Th22 cells and their canonical cytokines [27, 29, 31, 45]. Concomitant increases of Th17 and Th22 cells have been observed in CVB3 infection [28]. The decreased levels of these cells together were also described during HIV infection [45]. We also observed a positive correlation between the phenotype frequency of Th17 and Th22 in both HCMV + and HCMV- patients. The present study showed that reduced amounts of Th22 phenotype were concomitant with a reduction in Th17 cells.

Th17 cells and IL-17 increases have been reported in SOT transplant patients with HCMV active infection [46–48]. There is a controversy about the role of these cells against HCMV. It has been reported that these cells may have positive or negative effects on HCMV infection. The proinflammatory properties of IL-22 are enhanced when synergized with IL-17 in some viral infections [29, 44, 49]. In contrast, the simultaneous increase of Th22 and Th17 cells in CVB3 infection showed no development of IL-22 proinflammatory function, as neutralization of IL-22 exacerbated the disease severity by decreasing the IFN-γ cytokine and contributing to viral replication [28]. We did not examine the effect of IL-22 on HCMV; however, reduced levels of both cytokines (IL-22 and IL-17) in HCMV + patients and the restriction role of IL-22 against MCMV, as mentioned before, may bring the hypothesis that the synergistic function of these cytokines together could be in favor of antiviral responses in HCMV- patients.

Regarding the prediction potential of T cells in HCMV infection, most researchers have investigated other members of CD4^+^ T cells than recently identified Th22 cells. The reduced levels of Tfh and Th1 cells are associated with HCMV active infection and suggested for predicting HCMV outcomes in SOT transplant recipients [12–14, 18, 20, 24]. For example, Gerna et al. reported that the levels of IFN-γ producing CD4^+^ T cells of more than 0.4 cells/µl of blood in SOT patients were associated with protection from HCMV disease [50]. We showed that the cut-off value of ≤ 2.34% of the total Th22 phenotype could be used to predict HCMV infection, although we did not use virus-specific stimulation.

The reported data about the prediction value of Th17 cells showed that most studies could not find a proper association between viral outcomes and the frequency of these cells after transplantation [21, 51]. Just a study in 2018 showed that the pre-transplantation plasma levels of IL-23 cytokine, Th17-associated cytokine, more than 7 pg/ml might predict HCMV disease [52]. In our study, we showed that the phenotype frequency of Th17 cells with a cut-off value of ≤ 7.08% could be used to predict HCMV infection similar to Th22 after transplantation.

For the first time, the present study demonstrated the frequency of Th22 cells and production of IL-22 in HCMV infection among kidney transplant patients but with some limitations. First, we examined the frequency in a relatively small population. For better results, we need to extend the study population with different transplanted organs to investigate the effect of population diversity on the frequency of Th22 cells. Second, we did not have the baseline or samples before the initiation of viremia for our patients to investigate the frequency of Th22 cells before the onset of HCMV. So, working on such samples in future studies could help to find out if the low frequency of these cells is a cause or consequence of HCMV infection. Even, samples from the peak of infection or post-infection time points could give us information about the dynamics of Th22 cells during a course of infection. Third, our results represent the general frequency of these cells and their cytokines. Thus, to elucidate the specific responses, further analysis by HCMV-specific antigens or HCMV lysate stimulation would be helpful to determine correlation between Th22 cells and the virus. Lastly, to investigate the predicting value of Th22 cells in association with viral outcomes, their frequency should be investigated in patients with progressive infection or HCMV disease.

## Conclusion

In conclusion, the reduced level of Th22 cells and IL-22 cytokine in kidney recipients with active HCMV rather than the ones without infection, and a previous study determining the protective role of IL-22 against MCMV suggests the probability of their role in the immune response against HCMV which motivates further investigations. It seems that this reduction, along with reduced Th17 cells, might be a consequence of HCMV infection. These two cell types, Th22 and Th17, may have a synergistic function during HCMV infection. Moreover, it is possible to predict the occurrence of the infection by using the phenotype frequency of both Th22 and Th17 cells. Further investigations are essential to understand the precise function of Th22 cells against HCMV and the mechanism of their action.

## Materials and methods

### Study groups

This study was approved by the Shiraz University of Medical Sciences research ethics board and carried out at the Shiraz Transplant Research Center from 2018 to 2020. The study enrolled 20 seropositive kidney transplant recipients (D^+^/R^+^) from Abu-Ali Sina Hospital, Shiraz, Fars, Iran. Ten healthy individuals were also considered as control during the same period from the Iranian Blood Transfusion Organization, Shiraz, Fras, Iran. The healthy individuals did not use any medication at the time of sampling. Before sample collection, written informed consent was obtained from all subjects. Patients were categorized into two groups based on the result of quantitative real-time polymerase chain reaction (Real-time PCR). The first group consisted of 10 patients with active HCMV infection (reactivation) and a viral load above 10,000 copies/ml, considered the HCMV + group. The second group comprised 10 patients without active HCMV infection (HCMV-). All kidney transplant patients were under a daily standard triple immunosuppressive regimen, including two tablets (5 mg each) of prednisolone, one tablet (500 mg) of mycophenolate mofetil (CellCept), and two tablets (1 mg each) of tacrolimus (Prograf). At the time of sampling, none of the transplant groups (HCMV + and HCMV-) received antiviral therapy. Inclusion criteria were adults (≥ 18 years) transplanted for at least six months with discontinued antiviral prophylaxis. Patients co-infected with HCV, HBV, HIV, or BK virus, having second organ transplantation, or under HCMV prophylaxis or pre-emptive therapy were excluded from the study.

### Sample collection and processing

We collected blood samples from all subjects into EDTA tubes and transferred them to the laboratory within four hours. Plasma was collected after centrifugation from the upper layer and stored at -80 °C. The Peripheral Blood Mononuclear cells (PBMCs) were isolated by Ficoll density gradient centrifugation (Lymphodex, Inno-Train, Germany). These cells were preserved in a solution consisting of 90% heat-inactivated Fetal Bovine Serum (FBS) (Biochrom, Merck, Germany) and 10% Dimethyl Sulfoxide (DMSO) (Merck, Germany) and kept in liquid nitrogen (-196 °C) for further analysis.

### HCMV viral load quantification

A DNP extraction kit (Sinaclon, Iran) was used as instructed by the manufacturer to extract the HCMV DNA from plasma samples. GeneProof Cytomegalovirus PCR kit (Czechia) measured the HCMV viral load (the sensitivity is up to 122.594 IU/ml). The reaction mixture consisted of 15 µl of master mix, 1 µl of internal control, and 5 µl of cDNA in a total volume of 21 µl targeting the gene encoding the 4 IE antigens.

The real-time PCR was programmed in two subsequent steps by Step One Plus thermocycler (Applied Biosystems-Grand Island, USA). The first step consisted of one cycle, first at 37 °C for two minutes and then at 95 °C for 10 min as the initial denaturation. The second step consisted of forty-five cycles, first at 95 °C for 5 s, then at 60 °C for 40 s, and last at 72 °C for 20 s.

### HCMV IgG quantification

An ELISA kit (DIA.PRO, Milano, Italy) was used as instructed by the manufacturer to test the plasma samples for HCMV IgG. Briefly, 100 µl of diluted samples were dispensed into the wells coated with purified HCMV protein and incubated for 60 min at 37 °C. The wells are washed before the next step. Then, 100 µl enzyme conjugate was dispensed into each well and incubated for another 60 min at 37 °C. After the final wash, Chromogen/substrate and sulfuric acid were added in sequence, and the color intensity of each well was measured by Epoch Microplate Spectrophotometer (BioTek, Vermont, USA) at 450 nm against blanks. A calibration curve was established for quantitative determination using a set of calibrator solutions with concentrations of 0, 0.5, 1, 2, 4, and 8 IU/ml. A concentration higher than 0.5 IU/ml in each sample was considered positive for HCMV IgG.

### CD4^+^ T cells isolation

CD4^+^ T cells were negatively isolated from PBMCs by magnetic cell separation using a CD4^+^ T cell isolation kit and an LS column (Miltenyi Biotec, Bergisch Gladbach, Germany). The isolated cells were stained with PE CD3 (OKT3 clone, Cat. no., 317,307) and PerCp/Cy5.5 CD4 (OKT4 clone, Cat. no., 317,427) anti-human antibodies (Biolegend, USA). They were analyzed by flow cytometry, and a frequency of ≥ 95% CD3^+^ CD4^+^ T cells was considered pure isolated cells. Part of the purified CD4^+^ T cells was used to study gene expression by real-time PCR, and the rest of the cells were used to analyze the phenotype and cytokine profile of Th22 cells by flow cytometry.

### Flow cytometry

For surface staining, to analyze the Th22 phenotype, the CD4^+^ T cells were stained with PerCp/Cy5.5 CCR4 (L291H4 clone, Cat. no., 359,406), Alexa Flour 488 CCR6 (G034E3 clone, Cat. no., 353,414), and PE CCR10 (6588-5 clone, Cat. no., 341,503) anti-human antibodies (BioLegend, USA) in staining buffer (PBS + 1% FBS) for 10 min in the dark at 4 °C. The incubated cells were washed with 1 ml staining buffer and analyzed on a FACS Calibur analyzer (BD Pharmingen, San Jose, USA). Isotype controls of PerCp/Cy5.5 mouse IgG1, κ (MOPC-21 clone, Cat. no., 400,150), Alexa Flour 488 mouse IgG2b, κ (MPC-11 clone, Cat. no., 400,329), and PE Armenian hamster IgG (HTK888 clone, Cat. no., 400,907) were used for gating and antibody specificity (BioLegend, USA). The cells’ viability was determined by Propidium Iodide (Cat. no., P4170) (Sigma, Germany), and samples with ≥ 5% dead cells were excluded from the study.

For intracellular cytokine staining, cells were suspended at a density of 1 × 10^6^ cells/ml in complete RPMI 1640 (Biosera, France) medium supplemented with 10% FBS, 100 U/ml Penicillin-Streptomycin (Biosera, France), and 2 mM L-glutamine (Cat. no., 11,539,876) (Gibco). For cell stimulation, 2 µl of cell activation cocktail composed of Phorbol Myristate Acetate (PMA)/ionomycin (Cat. no., 423,301) (Biolegend, USA) was added to the medium, incubated under 5% CO_2_, at 37 °C, and in 95% humidity for six hours. Brefeldin A (Cat. no., 555,029) (Golgi Plug, BD Pharmingen, USA) was added for the final five hours of the incubation. Following the stimulation, cells were fixed in fixative buffer (1% paraformaldehyde) for 30 min in the dark at 4 °C, then washed twice in 2 ml permeabilization buffer consisting of 0.1% saponin (Cat. no., 84,510) (Sigma-Aldrich, Germany). For the determination of Th22 and Th17 cytokine profiles, cells were stained with PE IFN-γ (B27 clone, Cat. no., 506,506), FITC IL-17 A (BL168 clone, Cat. no., 512,303), and PerCp/Cy5.5 IL-22 (2G12A41 clone, Cat. no., 366,709) anti-human antibody (Biolegend, USA). Isotype control of PE mouse IgG1, κ (MOPC-21 clone, Cat. no., 400,112), FITC mouse IgG1, κ (MOPC-21 clone, Cat. no., 400,137), and PerCP/Cyanine5.5 mouse IgG2a, κ (MOPC-173 clone, cat. no., 400,251) were used for gating and antibody specificity (BioLegend, USA). Subsequently, the cells were analyzed on FACS Calibur and data analysis was performed by using FlowJo_V10 version 10.5.3 software (Tree Star Inc., Ashland, USA).

### Real-time PCR

To study the expression of the Th22 transcription factor, AHR, total RNA was extracted from the purified CD4^+^ T cells using RiboEx LS reagent (GeneAll, Seoul, Korea) according to the manufacturer’s instructions. Then, cDNA was generated using Reverse Transcription NG dART RT Kit (EURx, Gdańsk, Poland) with a combination of random hexamer and oligo(dT)20 primers. Real-time PCR was performed by adding 1 µl cDNA to 5 µl of SG qPCR Master Mix (2X) plus ROX (EURx, Gdańsk, Poland) with AHR specific primers. The mixture was incubated on Step One Plus thermocycler (Applied Biosystems-Grand Island, USA). The glyceraldehyde-3-phosphate dehydrogenase (GAPDH) housekeeping gene was used to normalize AHR expression, and the data were quantified by the 2^−ΔΔCT^ method. The real-time PCR condition and the sequence of primers are summarized in Table 2.

**Table 2:** The conditions of the Real-Time-PCR and the sequence of used primers

Real-Time PCR condition			
Steps	Temperature	Time	Number of cycles
Initial denaturation	95 °C	10 min^**a**^	1x^**b**^
Denaturation Annealing Extension	95 °C 60 °C 72 °C	15 s^**c**^ 30 s 30 s	40x
Primers			
AHR^**d**^	Forward: 5´AACATCACCTACGCCAGTCG3´ Reverse: 5´TGCCGCTTGGAAGGATTTGA3´		
GAPDH^**e**^	Forward: 5´GGACTCATGACCACAGTCC3´ Reverse: 5´CCAGTAGAGGCAGGGATGAT3´		

^**a**^min: minute; ^**b**^x: cycle; ^**c**^sec: second; ^**d**^AHR: aryl hydrocarbon receptor; ^**e**^GAPDH: glyceraldehyde-3-phosphate dehydrogenase.

### Statistical analysis

Data were presented as the mean ± Standard Error of the Mean (SEM). The data were analyzed using Kruskal-Wallis and one-way ANOVA, followed by the Tukey post host test, to compare more than two groups when appropriate (by the Shapiro-Wilk test). The unpaired t-test and Mann–Whitney U test were used to compare the two groups. The Spearman r correlation test was performed to determine correlation. Receiver Operating Characteristic (ROC) analysis was performed to calculate the most sensitive cut-off values. Data analysis was performed by GraphPad Prism 8 version 8.4.3 (GraphPad Software, San Diego, CA, USA) and IBM SPSS 26.0 software (SPSS Inc., Chicago, IL, USA). A p-value of ˂0.05 was considered statistically significant.

## Data Availability

The datasets used and analyzed during the current study are available from the corresponding author on reasonable request.

## List of Abbreviations

AHR: Aryl hydrocarbon receptor
CCR: C-C Chemokine Receptor
CVB3: Coxsackievirus B3
DMSO: Dimethyl sulfoxide
FBS: Fetal bovine serum
GAPDH: Glyceraldehyde-3-phosphate dehydrogenase
HCMV: Human cytomegalovirus
HCMV+: Patients with active HCMV infection
PBMC: Peripheral blood mononuclear cells
PMA: Phorbol myristate acetate
ROC: Receiver operating characteristic
SOT: Solid organ transplant
Th: T helper
